# Environmental risk assessment of pharmaceuticals at a seasonal holiday destination in the largest freshwater shallow lake in Central Europe

**DOI:** 10.1007/s11356-020-09747-4

**Published:** 2020-07-14

**Authors:** Eva Molnar, Gabor Maasz, Zsolt Pirger

**Affiliations:** Adaptive Neuroethology Research Group, Department of Experimental Zoology, MTA Centre for Ecological Research, Balaton Limnological Institute, Tihany, 8237 Hungary

**Keywords:** Environmental risk assessment,, Pharmacologically active compounds,, Ecotoxicological data,, Seasonal effects,, Touristic region,, Lake Balaton

## Abstract

The presence of pharmacologically active compounds (PhACs) in surface waters poses an environmental risk of chronic exposure to nontarget organisms, which is a well-established and serious concern worldwide. Our aim was to determine the temporal changes in ecological risk quotient (RQ) based on the concentrations of 42 PhACs from six sampling sites on seven sampling dates in the water of a freshwater lake in Central Europe preferentially visited by tourists. Our hypothesis was that the environmental risk increases during the summer holiday season due to the influence of tourists. Different experimental toxicological threshold concentrations and seasonal measured environmental concentrations of 16 PhACs were applied to ecological risk assessment. RQs of 4 dominant PhACs (diclofenac, estrone [E1], estradiol [E2], and caffeine) indicated high ecological risk (RQ > 1) for freshwater ecosystems. Additionally, our results confirmed the assumptions that the high tourist season had a significant impact on the calculated RQ; however, these results are mainly due to the concentration and temporal change of particular PhACs, including diclofenac (5.3-419.4 ng/L), E1 (0.1-5.5 ng/L), and E2 (0.1-19.6 ng/L). The seasonal dependent highest RQs changed as follows: 9.80 (June 2017; E2), 1.23 (August 2017; E1), 0.43 (November 2017; E1), 0.51 (April 2018; E1), 5.58 (June 2018, diclofenac), 39.50 (August 2018; diclofenac), and 30.60 (October 2018; diclofenac).

**Electronic supplementary material:**

The online version of this article (10.1007/s11356-020-09747-4) contains supplementary material, which is available to authorized users.

## Introduction 

Medicine has improved considerably in recent decades, contributing to the increase in the average age and fast growth of the human population. At the same time, the consumption of medication has changed significantly (Ginebreda et al. 2010; Guzel et al. 2019), resulted in an increased use of pharmaceuticals. However, wastewater treatment (WWT) technologies are not suitable for removing all kinds of pharmacologically active compounds (PhACs) with the same efficiency; therefore, a large majority of PhACs with their metabolites and conjugates have been appearing in all environmental compartments (surface waters, sediment, biota) worldwide (Halling-Sorensen et al. 1998; Kummerer 2004).

This is a concern for several reasons (Daughton and Ternes 1999; Diaz-Cruz et al. 2003). Information is lacking about possible harmful effects on nontarget freshwater organisms (e.g., zooplankton, molluscs, fish) when different PhACs form a mixture in receiving environments (Guzel et al. 2019). At the same time, it should also be noted that most measurement and risk assessment have been based on individual compound, but PhACs never occur as single substances in the environment. Therefore, to get a realistic picture about ecosystem involvement, investigation and assessment of multicomponent mixture effect of PhACs are required (De Zwart and Posthuma 2005; Lin et al. 2018; Heys et al. 2016). Additionally, the correct interpretation of measured environmental concentration (MEC) of PhACs is a big challenge for scientists, even today. Not only is the limited available experimental toxicity data (median effective concentration [EC50], median lethal concentration [LC50], and no observed effect concentration [NOEC]) a problem (Ginebreda et al. 2010; Hernando et al. 2006; la Farre et al. 2008; Thomaidi et al. 2015), but even if such data exist and are accessible, they are usually described based on different observations (e.g., various endpoints and species), so, in other words, they are not consistent (Lange and Dietrich 2002). Of course, this is understandable because different studies of PhACs have been conducted in vivo using different mechanisms; therefore, the effect of the given PhACs has been observed using different endpoints (e.g., growth, mortality, reproduction or developmental, behavioural effects, and molecular, cellular, tissue level changes). Even though the MEC is known, since there is a lack of standardized experimental toxicity data in many cases (la Farre et al. 2008; Thomaidi et al. 2015), the ecological risk assessment (ERA) cannot be appropriately performed (Ferrari et al. 2004).

To estimate the harmful effect of PhACs on an ecosystem, a risk quotient (RQ) is usually applied, which is defined as the ratio of the maximum MEC to the predicted no effect concentrations (PNEC), where PNEC depends on the available toxicological data (Carlsson et al. 2006; Deo 2014; Ferrari et al. 2004; Hernando et al. 2006; Komori et al. 2013). To get the most realistic ecological RQ values, PNECs need to be derived from species sensitivity distribution (SSD) curve (Posthuma et al. 2002) or at least experimental NOEC, or E(L)C50. Other PNECs estimated based on, for example, ECOSAR (Sanderson et al. 2004) are only used for cases which no laboratory data are available; however, they need to be managed with a high degree of uncertainty.

In other aspects, the degree of risk depends on the concentration data and the forms and migration of PhACs in the environmental elements, and these levels are influenced by among other factors, the efficiency of the WWT technology applied and the resistance of (bio)degradation, complexation, sorption, bioaccumulation, defined daily doses, dosage of medicine (periodical or continuous), and even weather conditions (Andreozzi et al. 2002; Bouissou-Schurtz et al. 2014). Furthermore, for a comprehensive ERA, all environmental elements should be examined because PhACs, depending on the environmental conditions (e.g., temperature, UV radiation), are distributed between different matrices (water, sediment, suspended solid, biofilm) (Dobor et al. 2012). Besides environmental conditions, the effect of tourism also needs to be considered for ERA. The improving tourism industry frequently poses a risk to the ecosystems by the increased load of WWT plant locally and many recreational activities (e.g., swimming, sailing, kayaking, canoeing, diving, or fishing), respectively (Hadwen et al. 2005; Katircioglu 2014; Mihalic 2000). Increased PhAC levels, also including recreational substances (e.g., caffeine and illicit drugs), during high tourism season is a well-known phenomenon (Guzel et al. 2019; Lin et al. 2018; Nakada et al. 2017; Zhang et al. 2017) in rivers flowing throughout cities; however, there are only limited data in case of lakes (Maasz et al. 2019). Based on all of them, the production of an accurate and definite assessment of risk level is a very difficult and complex task; however, approximate calculations are also necessary and useful to prevent environmental damage.

This study complements and uses another approach to analyse our earlier screening data resulted from investigating the presence of 134 PhACs in the surface water of Lake Balaton and its catchment area from June 2017 to April 2018. Taking the studied period and sampled sites belonging to the lake into account, 39 PhACs were detected and quantified in water samples from the lake (Maasz et al. 2019). This was the first extended qualitative and quantitative study to present data on the occurrence of PhACs derived from several chemical classes in this lake. Measurements have continued, and the database has been complemented with further MEC data from June, August, and October 2018. In total, it was possible to consider the ERAs of 42 PhACs. The main goals of the present study were to estimate the environmental risk of single and mixed PhACs in the surface water of Lake Balaton, a popular touristic region in Europe, subsequently, to explore a possible correlation between the magnitude of the actual hazard and impacts of seasonal changes (spring, summer, autumn, winter).

## Experimental methodology

### Study area

**Fig. 1:**
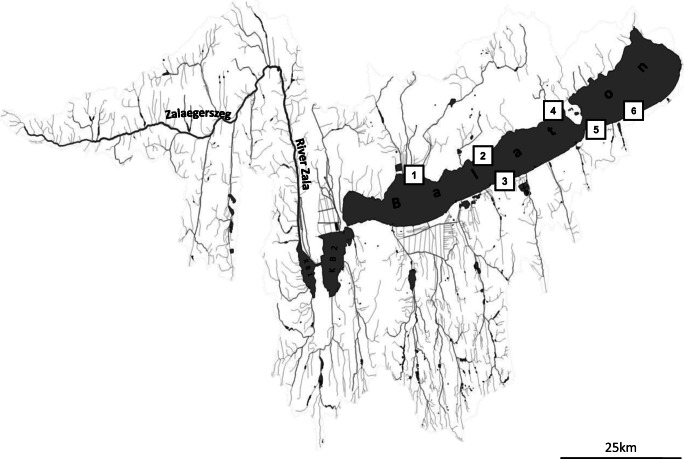
Hydrogeography of Lake Balaton. The positions marked from 1 to 6 belong to the near-coastal area of the lake. The sampling points (by coordinates) are as follows: 1 Szigliget (46.78541, 17.4349), 2 Révfülöp (46.82411, 17.60672), 3 Balatonlelle (46.79708, 17.72528), 4 Tihany-Sajkod (46.90339, 17.85037), 5 Zamárdi (46.88525, 17.93139), and 6 Siófok (46.91102, 18.04604)

The study was conducted in Lake Balaton (Fig. 1), which is one of the largest (A, 594 km^2^; mean depth, 3.2 m; V, ~ 1.8 km^3^) freshwater shallow lakes in Central Europe (Hungary) (Istvanovics et al. 2007) and very popular with tourists. The Lake Balaton resort area is an internationally important tourist and recreation centre visited by millions of tourists a year, especially in summer season (Maasz et al. 2019; URL1 2019). The maximum number of guest nights at commercial accommodation in the counties surrounding Lake Balaton approaches ~ 900,000 in an average summer month (e.g., August) in a high tourist season also in 2017 and 2018, while this value is only ~ 300,000 in winter (see Supplementary Fig. 1). The human population shows unequal spatiotemporal distribution in this region; two-thirds of the local resident population (~ 380,000 people) inhabit the near-coastal area of the lake (URL1 2019; URL2 2020). Nowadays, more than 40 WWT plants are being situated in the catchment area of Lake Balaton; the largest one (with a capacity of 50,000 m^3^/day) can be found in Zalaegerszeg (URL3 n.d.) which is the largest town of the catchment area (with ~ 60,000 inhabitants) (URL1 2019). This town is located on the riverbank of River Zala (the largest tributary of Lake Balaton) supplying ~ 50% of the lake’s total surface water input (URL3 n.d.). Since the wastewater effluent reaches directly the River Zala, it also plays a potential role in the PhACs pollution of Lake Balaton.

### Sample collection, preparation, and measurement

The designation of sampling sites (Fig. 1) was based on our previous study (Maasz et al. 2019), and the current research may be considered to be the continuation of that work. Forty-two water samples used for the present study were collected in June, August, and November of 2017 and April, June, August, and October of 2018 from six sampling sites on the littoral region of the lake (see Supplementary Table 1).

All water samples were collected by a water-column sample device from the middle of the water level in 2-L amber silanized glass bottles with Teflon-faced caps. One litre of each sample was acidified by applying 100% formic acid (due to sorbent type compatibility) to pH 3.5–4.0. Internal standards (Citalopram-d6, Carbamazepine-d10, E2-13C3, and N-ethyloxazepam) were added to samples before filtration; the final concentration was 5 ng/L for each standard, and these were used for the quantification of samples. After spiking by internal standards, samples were vacuum filtered through a GF/F 0.7-μm glass microfibre filter (#516-0345, VWR). The solid phase extraction (SPE) of samples was implemented using AutoTrace 280-automated SPE system (Thermo Scientific). SPE extracts were evaporated using an inert nitrogen gas stream. Analytical measurements and detection were performed using an ACQUITY UPC2 supercritical fluid chromatography system (Waters) coupled with a Xevo TQ-S Triple Quadrupole Mass Spectrometer (Waters). Data were recorded by MassLynx software (V4.1 SCN950) and evaluated by TargetLynx XS software. The details of analytical measurements with validation parameters of measured PhACs and data evaluation are published in our previous paper (Maasz et al. 2019).

### Calculation of ERA

ERA is based on ecotoxicological threshold data from experiments on aquatic organisms (algae, Cladocera [usually *Daphnia* sp.], and/or fish species). Accordingly, E(L)C50 and NOEC values derived from acute and chronic tests, respectively, are taken into consideration. Applying them, the SSD curve and the hazard concentrations (e.g., HC5, which 5% of the species in the SSD exhibit an effect; Supplementary Fig. 2) are also determined by Chemical Aquatic Fate and Effects (CAFE) database and software (Bejarano et al. 2016). Using these data, the PNEC is calculated (Eq. 1) as the ratio of the E(L)C50, NOEC, or HC5 data and an assessment factor (AF);

1 $$ PNEC=\frac{\ \mathrm{E}\left(\mathrm{L}\right)\mathrm{C}50\ \mathrm{or}\ \mathrm{NOEC}\ \mathrm{or}\ \mathrm{HC}5}{\mathrm{AF}} $$

The magnitude of the AF depends on the available toxicological information. The reliability of the results increases if toxicological data for aquatic organisms are available at multiple different trophic levels. Hence, the value of AF is decreased in cases of large and relevant datasets. For example, if toxicity data are only available based on E(L)C50, an AF of 1000 is used, but when NOEC is derived from experiments with a single trophic level (e.g., fish), an AF of 100 is applied, and if NOEC for two trophic levels are available (e.g., fish and Cladocera), AF = 50 is used. If NOECs are known for all three trophic levels, then AF is equal to 10 (Hamre 2006). In case of using at least five different species (independently on trophic levels) with the same toxicological data, meaning the HC5 value is known, AF = 5 (Amiard and Amiard-Triquet 2015).

If different toxicity data are available for each trophic level, the lowest concentration limit results will be used to determine PNEC, as ERA is based on the most sensitive elements of the ecosystem, in order to estimate ecological hazard for the worst-case scenario (Thomaidi et al. 2015).

If no experimental toxicological data are available, then predicted E(L)C50 values from the US Environmental Protection Agency Ecological Structure Activity Relationships Class Program (ECOSAR database) are usually used (Sanderson et al. 2004); however, the data from this database are highly uncertain; therefore, the applicable is AF = 1000 (Zhang et al. 2017).

ERA characterization is possible after the measurement of environmental concentrations and determination of the toxicology threshold values of investigated pollutants, because RQ, which is used to categorize harmful effects for the ecosystem, is defined as the ratio of the maximum MEC to the PNEC (Eq. 2):

2 $$ RQ=\frac{\mathrm{MEC}}{\mathrm{PNEC}} $$

In general, RQ < 0.01 denotes a negligible risk, RQ < 0.1 reveals a low risk, 0.1 < RQ < 1 represents a medium risk, and RQ > 1 indicates a high ecological risk to aquatic organisms (Ma et al. 2016; EU Commission 2003).

The following method was used to track risk levels over time. From the six sampling sites (Fig. 1), the highest MEC was selected for each PhAC and investigated month. Their maximum RQ values among six sampled sites were defined as the maxRQ. From the highest maxRQ, each sampled month was determined, termed maxRQperiod; this is independent of the kind of PhAC and its relationship over time can be studied. When the highest maxRQs were calculated for the whole studied period, separately for each PhAC, we generally define this value as MAX RQ values. Based on MAX RQs, the different level of risk (high, medium, low, and negligible) for each PhACs can be determined in the whole investigation period (see Supplementary Table 2).

In the vast majority of aquatic mixture toxicity studies, the toxicity of a mixture is assessed by concentration addition (CA) model and neglected the toxic modes of action of the mixture constituents. The CA model implies that the contribution of the individual toxicants to the overall effect can be added in the form of toxic units (TU). The CA of a mixture can be described by the following equation (De Zwart and Posthuma 2005) with slight modifications:

3 $$ TU=\sum \limits_{i=1}^n\frac{MEC_i}{E(L)C{50}_i\  or\ {NOEC}_i} $$

where MEC_i_ is the actual concentrations and E(L)C50_i_ or NOEC_i_ is the exposure concentrations of a given PhAC that cause the same standard toxicological response for all compounds. The TU is a dimensionless expression. It has only one threshold; if its value is greater than 1, it implies a potential risk.

## Results and discussion

### Seasonal changes in PhACs concentration and ERA

**Fig. 2:**
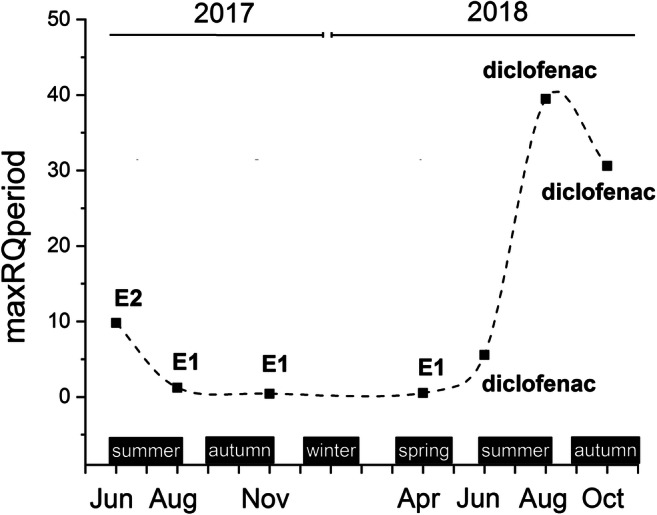
Seasonal fluctuation of maxRQperiods in Lake Balaton in the investigated months (striped, summer seasons; dashed vertical, autumn seasons; gridded, winter season; waved, spring season). E1, estrone; E2, estradiol

New PhACs, theophylline (28.9–59.6 ng/L), barbital (94.8 ng/L), and diclofenac (5.3–419.4 ng/L) (see detailed in Supplementary Table 1) were detected in the lake in addition to the 39 compounds published earlier (Maasz et al. 2019). The collection of the necessary raw predicted and/or experimental toxicological data (E(L)C50, NOEC, and HC5) and the determination of AF and PNEC values of 42 PhACs, summarized in Table 1, were essential to perform ERA. Table 1 contains various PNEC values in case of some PhACs. For example, 6 different PNECs were calculable in a range of 0.1–44.0 in the case of E2 from available ecotoxicological data. However, if the data collection is not sufficiently thorough and the selection method among them is not appropriate (e.g., ECOSAR is applied instead of available laboratory data, or acute experimental results are used in place of known chronic outcomes), the ERA will also be wrong even in orders of magnitude. Since the experimental toxicological data and realistic PNEC values were found only in case of 16 PhACs from 42, ERA and seasonal fluctuation of RQs were emphasized to these compounds in this study. Table 2 shows the results of the ERA (based on RQ values) calculated from MEC and the PNEC data. The highest RQ values in the months investigated (maxRQperiod) were as follows: 9.80 (June 2017; E2), 1.23 (August 2017; E1), 0.43 (November 2017; E1), 0.51 (April 2018; E1), 5.58 (June 2018, diclofenac), 39.50 (August 2018; diclofenac), and 30.60 (October 2018; diclofenac). Therefore, based on these results, we concluded that the values of maxRQperiod varied seasonally. The seasonal fluctuation of maxRQperiod was plotted and displayed in Fig. 2; this is the first study to present such investigation in freshwater lakes. This fluctuation in our study area was caused by changes in the presence and concentration of E1, E2, and diclofenac especially. The risk of these PhACs presented was typically higher during the summer seasons (e.g., caffeine, 1.16; E2, 9.80; and E1, 5.52 in June or August) than in any other months investigated (e.g., caffeine, 0.00 [< LOQ]; E2, 0.00 [< LOQ], and E1, 0.43 in November). Similar season-influenced phenomena in detected environmental concentration values of recreational substances (e.g., illicit drugs) have already been observed in Lake Balaton by our research group (Maasz et al. 2019), and the occurrence and concentration of other PhACs (e.g., methamphetamine, amphetamine, ketamine, and ephedrine) have been also reported in the urban rivers of Beijing in China (Zhang et al. 2017). The frequency of occurrence and levels of several PhACs (e.g., carbamazepine, caffeine, citalopram, and diclofenac) have also been found to differ by season in River Ceyhan in Turkey (Guzel et al. 2019) and Xiangjiang River in China (Lin et al. 2018).

**Table 1:** Raw toxicological data for the 42 investigated PhACs. Ecotoxicological data are collected from ECOSAR (Sanderson et al. 2004) and/or CAFE database and/or several papers (see references), with their AF and calculated PNECs in ng/L (n.d. = no data)

PhACs	Ecotoxicological data							AF	PNEC	Ref.
	Based on acute test results			Based on chronic test result			Based on SSD			
	E(L)C_50_ (algae)	E(L)C_50_ (Cladocera)	E(L)C_50_ (fish)	NOEC (algae)	NOEC (Cladocera)	NOEC (fish)	HC5			
	[ng/L]								[ng/L]	
Alprazolam	6.28E + 05	5.08E + 05	5.41E + 06	n.d.	n.d.	n.d.	n.d.	1.00E + 03	5.08E + 02	Sanderson et al. 2004
Atropine	2.66E + 06	6.64E + 06	2.00E + 07	n.d.	n.d.	n.d.	n.d.	1.00E + 03	2.66E + 03	Sanderson et al. 2004
Barbital	n.d.	n.d.	1.16E + 09	n.d.	n.d.	n.d.	n.d.	1.00E + 03	1.16E + 06	Sanderson et al. 2004
Benzoylecgonine	1.20E + 10	6.81E + 09	3.35E + 10	n.d.	n.d.	n.d.	n.d.	1.00E + 03	6.81E + 06	Mendoza et al. 2014
Bisoprolol	3.15E + 06	8.20E + 06	1.13E + 08	n.d.	n.d.	n.d.	n.d.	1.00E + 03	3.15E + 03	Sanderson et al. 2004
Bupropion	3.30E + 06	9.50E + 05	3.30E + 07	n.d.	n.d.	n.d.	n.d.	1.00E + 03	9.50E + 02	Vestel et al. 2016
Buspirone	2.60E + 06	5.16E + 06	6.70E + 07	n.d.	n.d.	n.d.	n.d.	1.00E + 03	2.60E + 03	Sanderson et al. 2004
**Caffeine**	6.85E + 06	4.70E + 07	8.05E + 08	n.d.	n.d.	n.d.	n.d.	1.00E + 03	6.85E + 03	Sanderson et al. 2004
	n.d.	n.d.	n.d.	n.d.	1.20E + 02	n.d.	n.d.	1.00E + 02	1.20E + 00	Lu et al. 2013
	n.d.	n.d.	n.d.	n.d.	n.d.	n.d.	1.16E + 04	5.00E + 00	**2.32E + 03**	CAFE
**Carbamazepine**	8.15E + 06	6.36E + 06	1.40E + 07	n.d.	n.d.	n.d.	n.d.	1.00E + 03	6.36E + 03	Sanderson et al. 2004
	n.d.	n.d.	n.d.	1.00E + 06	n.d.	n.d.	n.d.	1.00E + 01	**1.00E + 04**	Zhang et al. 2012
	n.d.	n.d.	n.d.	n.d.	1.00E + 05	n.d.	n.d.			Lurling et al. 2006
	n.d.	n.d.	n.d.	n.d.	n.d.	1.78E + 06	n.d.			Madureira et al. 2011
**Citalopram**	7.29E + 05	6.35E + 05	6.88E + 06	n.d.	n.d.	n.d.	n.d.	1.00E + 03	6.35E + 02	Sanderson et al. 2004
	n.d.	n.d.	n.d.	n.d.	n.d.	1.00E + 03	n.d.	1.00E + 02	**1.00E + 01**	Olsen et al. 2014
**Clozapine**	1.47E + 06	2.15E + 06	2.60E + 07	n.d.	n.d.	n.d.	n.d.	1.00E + 03	1.47E + 03	Sanderson et al. 2004
	n.d.	n.d.	n.d.	n.d.	n.d.	2.85E + 04	n.d.	1.00E + 02	**2.85E + 02**	Nallani, et al. 2016
Cocaine	2.28E + 06	4.91E + 06	1.30E + 07	n.d.	n.d.	n.d.	n.d.	1.00E + 03	2.28E + 03	Sanderson et al. 2004
**Diazepam**	1.42E + 06	2.26E + 06	2.80E + 07	n.d.	n.d.	n.d.	n.d.	1.00E + 03	1.42E + 03	Sanderson et al. 2004
	n.d.	n.d.	n.d.	n.d.	n.d.	2.60E + 05	n.d.	1.00E + 02	**2.60E + 03**	Oggier et al. 2010
**Diclofenac**	7.71E + 06	4.24E + 06	4.94E + 06	n.d.	n.d.	n.d.	n.d.	1.00E + 03	4.24E + 03	Sanderson et al. 2004
	n.d.	n.d.	n.d.	n.d.	n.d.	n.d.	n.d.	n.d.	5.00E + 01	EU Commission JRC et al. 2018
	n.d.	n.d.	n.d.	n.d.	n.d.	1.06E + 03	n.d.	1.00E + 02	**1.06E + 01**	Schwaiger et al. 2004
**E1**	1.66E + 06	5.60E + 05	7.40E + 04	n.d.	n.d.	n.d.	n.d.	1.00E + 03	7.40E + 01	Sanderson et al. 2004
	n.d.	n.d.	n.d.	n.d.	n.d.	n.d.	n.d.	n.d.	3.60E + 00	EU Commission JRC et al. 2018
	n.d.	n.d.	n.d.	n.d.	n.d.	1.00E + 02	n.d.	1.00E + 02	**1.00E + 00**	Dammann et al. 2011
**E2**	8.00E + 05	2.77E + 05	4.40E + 04	n.d.	n.d.	n.d.	n.d.	1.00E + 03	4.40E + 01	Sanderson et al. 2004
	n.d.	n.d.	n.d.	n.d.	n.d.	n.d.	n.d.	n.d.	4.00E **-** 01	EU Commission JRC et al. 2018
	n.d.	n.d.	n.d.	8.00E + 04	n.d.	n.d.	n.d.	1.00E + 01	1.00E **-** 01	Julius et al. 2007
	n.d.	n.d.	n.d.	n.d.	1.00E + 02	n.d.	n.d.			Marcial et al. 2003
	n.d.	n.d.	n.d.	n.d.	n.d.	1.00E + 00	n.d.			Routledge et al. 1998; Lahnsteiner et al. 2006
	n.d.	n.d.	n.d.	n.d.	n.d.	n.d.	n.d.	n.d.	7.30E **-** 01	Wu et al. 2014
	n.d.	n.d.	n.d.	n.d.	n.d.	n.d.	1.00E + 01	5.00E + 00	**2.00E + 00**	CAFE
**E3**	4.39E + 06	1.45E + 06	1.50E + 04	n.d.	n.d.	n.d.	n.d.	1.00E + 03	1.50E + 01	Sanderson et al. 2004
	n.d.	n.d.	n.d.	n.d.	n.d.	4.65E + 01	n.d.	1.00E + 02	**4.65E - 01**	Lei et al. 2014
**EE2**	6.77E + 05	2.34E + 05	4.00E + 04	n.d.	n.d.	n.d.	n.d.	1.00E + 03	4.00E + 01	Sanderson et al. 2004
	n.d.	n.d.	n.d.	n.d.	n.d.	4.40E + 01	n.d.	1.00E + 02	**4.40E - 01**	Kristensen et al. 2005
**Fluoxetine**	3.45E + 05	1.78E + 05	1.72E + 06	n.d.	n.d.	n.d.	n.d.	1.00E + 03	1.78E + 02	Sanderson et al. 2004
	n.d.	n.d.	n.d.	n.d.	n.d.	5.40E + 04	n.d.	5.00E + 01	**1.08E + 03**	Mennigen et al. 2010
	n.d.	n.d.	n.d.	7.20E + 04	n.d.	n.d.	n.d.			DeLorenzo and Fleming 2008
Ketamine	8.61E + 05	1.07E + 06	1.30E + 07	n.d.	n.d.	n.d.	n.d.	1.00E + 03	8.61E + 02	Sanderson et al. 2004
**Lamotrigine**	n.d.	n.d.	n.d.	n.d.	n.d.	1.50E + 10	n.d.	1.00E + 02	**1.50E + 08**	Deo 2014
Levonorgestrel	2.28E + 06	1.31E + 06	5.56E + 05	n.d.	n.d.	n.d.	n.d.	1.00E + 03	5.56E + 02	Sanderson et al. 2004
Lidocaine	2.61E + 06	7.52E + 06	1.07E + 08	n.d.	n.d.	n.d.	n.d.	1.00E + 03	2.61E + 03	Sanderson et al. 2004
Losartan	n.d.	n.d.	n.d.	n.d.	n.d.	n.d.	n.d.	n.d.	1.90E + 03	Helwig et al. 2016
MDMA	2.30E + 06	2.16E + 05	2.42E + 07	n.d.	n.d.	n.d.	n.d.	1.00E + 03	2.16E + 02	Mendoza et al. 2014
Methadone	4.12E + 07	3.81E + 07	1.10E + 08	n.d.	n.d.	n.d.	n.d.	1.00E + 03	3.81E + 04	Sanderson et al. 2004
**Metoprolol**	n.d.	n.d.	n.d.	n.d.	6.15E + 06	n.d.	n.d.	1.00E + 02	**6.15E + 04**	Dzialowski et al. 2006
Midazolam	4.65E + 05	2.89E + 05	2.90E + 06	n.d.	n.d.	n.d.	n.d.	1.00E + 03	2.89E + 02	Sanderson et al. 2004
Mirtazapine	n.d.	n.d.	n.d.	n.d.	n.d.	n.d.	n.d.	n.d.	3.20E + 04	Helwig et al. 2016
Naproxen	2.30E + 07	1.51E + 07	2.43E + 07	n.d.	n.d.	n.d.	n.d.	1.00E + 03	1.51E + 04	Sanderson et al. 2004
Nordiazepam	1.19E + 06	1.71E + 06	2.10E + 07	n.d.	n.d.	n.d.	n.d.	1.00E + 03	1.19E + 03	Sanderson et al. 2004
Olanzapine	1.41E + 08	n.d.	n.d.	n.d.	n.d.	n.d.	n.d.	1.00E + 03	1.41E + 05	Jiahua 2015
Perindopril	n.d.	n.d.	n.d.	n.d.	n.d.	n.d.	n.d.	n.d.	9.90E + 05	Webb 2001
**Progesterone**	3.30E + 06	1.00E + 06	7.33E + 05	n.d.	n.d.	n.d.	n.d.	1.00E + 03	7.33E + 02	Sanderson et al. 2004
	n.d.	n.d.	n.d.	n.d.	1.00E + 05	n.d.	n.d.	1.00E + 02	**1.00E + 03**	Kashian and Dodson 2004
**Quetiapine**	n.d.	n.d.	n.d.	n.d.	n.d.	1.00E + 05	n.d.	1.00E + 01	**1.00E + 04**	AstraZeneca
**Testosterone**	2.90E + 06	1.70E + 06	1.43E + 06	n.d.	n.d.	n.d.	n.d.	1.00E + 03	1.43E + 03	Sanderson et al. 2004
	n.d.	n.d.	n.d.	n.d.	1.00E + 05	n.d.	n.d.	1.00E + 02	**1.00E + 03**	Clubbs and Brooks 2007
Tetracaine	7.45E + 05	1.36E + 06	2.20E + 06	n.d.	n.d.	n.d.	n.d.	1.00E + 03	7.45E + 02	Sanderson et al. 2004
Theophylline	9.70E + 06	1.00E + 06	1.68E + 09	n.d.	n.d.	n.d.	n.d.	1.00E + 03	1.00E + 03	Sanderson et al. 2004
Tiapride	8.72E + 06	4.80E + 07	7.89E + 08	n.d.	n.d.	n.d.	n.d.	1.00E + 03	8.72E + 03	Sanderson et al. 2004
Tramadol	1.04E + 06	3.20E + 04	7.72E + 06	n.d.	n.d.	n.d.	n.d.	1.00E + 03	3.20E + 01	Sanderson et al. 2004
Verapamil	n.d.	n.d.	3.60E + 07	n.d.	n.d.	n.d.	n.d.	1.00E + 03	3.60E + 04	Sanderson et al. 2004
Zolpidem	6.35E+05	5.19E+05	5.54E+06	n.d.	n.d.	n.d.	n.d.	1.00E + 03	5.19E + 02	Sanderson et al. 2004

**Table 2:** MEC data (in ng/L), calculated maxRQ, maxRQperiod, and MAX RQ values of PhACs, as well as risk levels of Lake Balaton in the seven investigated periods (LOQ = limit of quantitation)

PhACs	Lake Balaton (1-6)															
	June 2017		August 2017		November 2017		April 2018		June 2018		August 2018		October 2018		June 2017 - October 2018	
	MEC	maxRQ	MEC	maxRQ	MEC	maxRQ	MEC]	maxRQ	MEC	maxRQ	MEC	maxRQ	MEC	maxRQ	MAX RQ	Level of risk
	[ng/L]		[ng/L]		[ng/L]		[ng/L]		[ng/L]		[ng/L]		[ng/L]			
Diclofenac	< LOQ	-	< LOQ	-	< LOQ	-	< LOQ	-	5.91E + 01	5.58E + 00	4.19E + 02	3.95E + 01	3.24E + 02	3.06E + 01	3.95E + 01	High
E2	1.96E + 01	9.80E + 00	2.00E - 01	1.00E - 01	< LOQ	-	1.95E - 01	9.75E - 02	3.00E + 00	1.50E + 00	< LOQ	-	6.50E - 02	3.25E - 02	9.80E + 00	High
E1	5.52E + 00	5.52E + 00	1.23E + 00	1.23E + 00	4.30E - 01	4.30E - 01	5.10E - 01	5.10E - 01	< LOQ	-	1.81E + 00	1.81E + 00	4.25E - 01	4.25E - 01	5.52E + 00	High
Caffeine	< LOQ	-	8.99E + 01	3.88E - 02	< LOQ	-	< LOQ	-	1.39E + 03	6.00E - 01	2.68E + 03	1.16E + 00	2.42E + 03	1.04E + 00	1.16E + 00	High
EE2	< LOQ	-	< LOQ	-	1.80E - 01	4.09E - 01	< LOQ	-	< LOQ	-	< LOQ	-	< LOQ	-	4.09E - 01	Medium
E3	1.00E - 01	2.15E - 01	1.30E - 01	2.80E - 01	< LOQ	-	< LOQ	-	< LOQ	-	< LOQ	-	< LOQ	-	2.80E - 01	Medium
Citalopram	1.30E - 01	1.30E - 02	2.00E - 01	2.00E - 02	< LOQ	-	2.44E + 00	2.44E - 01	< LOQ	-	< LOQ	-	< LOQ	-	2.44E - 01	Medium
Carbamazepine	6.88E + 01	6.88E - 03	4.63E + 01	4.63E - 03	1.59E + 01	1.59E - 03	7.75E + 01	7.75E - 03	1.45E + 01	1.45E - 03	1.66E + 01	1.66E - 03	2.41E + 01	2.41E -03	7.75E - 03	Negligible
Clozapine	5.40E - 01	1.89E - 03	5.50E - 01	1.93E - 03	< LOQ	-	< LOQ	-	< LOQ	-	< LOQ	-	5.54E - 01	1.94E - 03	1.94E - 03	Negligible
Fluoxetine	1.68E + 00	1.56E - 03	< LOQ	-	< LOQ	-	< LOQ	-	< LOQ	-	< LOQ	-	< LOQ	-	1.56E - 03	Negligible
Progesterone	9.60E - 01	9.60E - 04	1.31E + 00	1.31E - 03	< LOQ	-	1.13E + 00	1.13E - 03	< LOQ	-	< LOQ	-	< LOQ	-	1.31E - 03	Negligible
Testosterone	< LOQ	-	< LOQ	-	< LOQ	-	1.09E + 00	1.09E - 03	< LOQ	-	< LOQ	-	< LOQ	-	1.09E - 03	Negligible
Diazepam	< LOQ	-	< LOQ	-	< LOQ	-	2.50E - 01	9.62E - 05	< LOQ	-	< LOQ	-	< LOQ	-	9.62E - 05	Negligible
Metoprolol	< LOQ	-	5.08E + 00	8.26E - 05	< LOQ	-	1.17E + 00	1.90E - 05	2.64E - 01	4.28E - 06	< LOQ	-	1.25E + 00	2.04E - 05	8.26E - 05	Negligible
Quetiapine	1.20E - 01	1.20E - 05	1.10E - 01	1.10E - 05	< LOQ	-	< LOQ	-	< LOQ	-	< LOQ	-	< LOQ	-	1.20E - 05	Negligible
Lamotrigine	8.57E + 00	5.71E - 08	1.62E + 02	1.08E - 06	2.21E + 01	1.47E - 07	3.34E + 01	2.23E - 07	< LOQ	-	< LOQ	-	5.54E + 01	3.69E - 07	1.08E - 06	Negligible
**maxRQperiod**	**9.80E + 00**		**1.23E + 00**		**4.30E - 01**		**5.10E - 01**		**5.58E + 00**		**3.95E + 01**		**3.06E + 01**

Regarding the contamination input aspect of surface water, the environmental concentrations of PhACs vary depending on their chemical stability, biodegradability, physicochemical characteristics, and the efficiency of WWT technology (Bouissou-Schurtz et al. 2014). For example, microbiological activity is influenced by temperature during WWT, as the efficiency of bacterial removal decreases in winter (Couto et al. 2019). Climate effects (e.g., temperature, ultraviolet exposure, rainfall, wind) can also modify the measured concentration of PhACs at the investigated sites (Zhang et al. 2017). Moreover, the change of season affects tourists; thereby the spatial distribution of the population and, as consumption and excretion of PhACs contribute to the detected contamination, the impact of tourism cannot be neglected. Additionally, the typical health problems and the most consumed PhACs change depending on weather conditions and season. For some PhACs, seasonal consumption patterns were also observed; for example, some antipyretics (e.g., diclofenac, ibuprofen, and naproxen) have higher usage rates during winter than spring, summer, or autumn. At the same time, similar to our observations in this study, other PhACs such as carbamazepine showed a similar presence in all seasonal periods (Camacho-Munoz et al. 2014; Couto et al. 2019). Consequently, the season-influenced phenomenon of PhACs is the outcome of a very difficult, complex, and multifactor process.

As Table 2 indicates, based on our MAX RQ data, 4 PhACs in Lake Balaton were > 1 including diclofenac (39.50), E2 (9.80), E1 (5.52), and caffeine (1.16), indicating high ecological risk for freshwater ecosystems. Another 3 PhACs received a medium (EE2 [0.41], E3 [0.28], citalopram [0.24]) classification, and the remaining 9 were negligible. A study collecting the PhACs concentrations in European surface waters and performing ERA has already reported high risk levels in case of all 7 compounds, although the standard method of calculating ERA based on maximal MECs results in overestimation of the actual risk levels. To avoid overestimation, updated RQs can be assessed considering the frequency that MECs exceed PNECs and using mean MECs instead of maximal MECs (Zhou et al. 2019). Our data were also investigated using this improved method; the updated ERA results showed that the risk of PhACs decreases at least one level compared with MAX RQs (data not shown); however, seasonal effects can be better observed considering the maxRQperiod values presented in this paper.

**Fig. 3:**
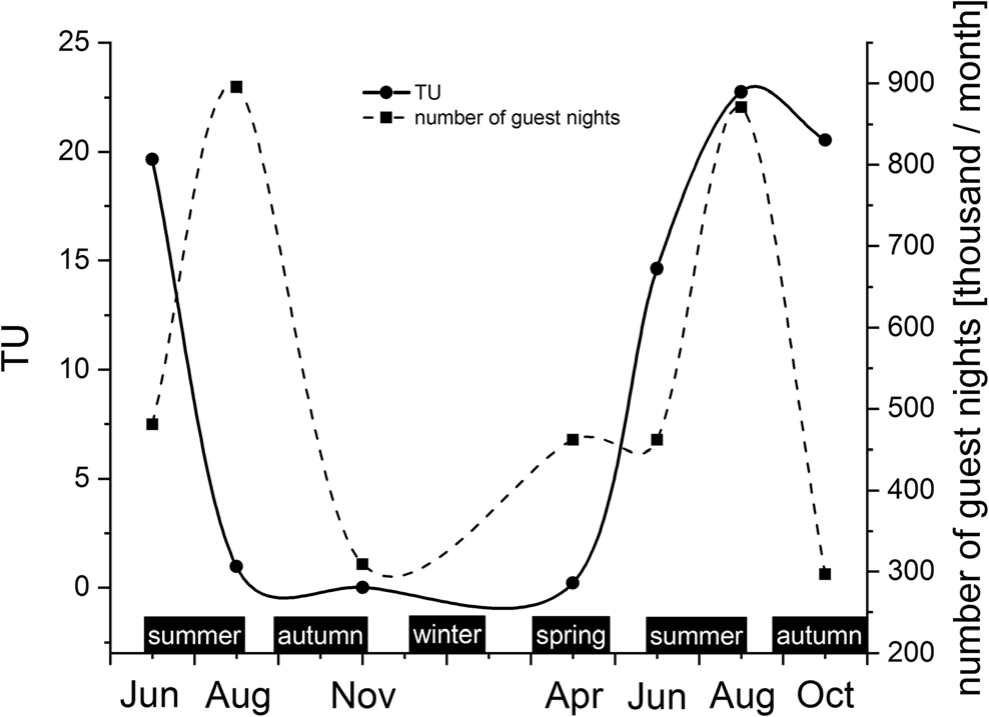
Seasonal fluctuation of TU and number of guest nights in Lake Balaton in the investigated months

The mixture effect of the examined 16 PhACs was estimated based on their NOEC levels. The characteristic shape of the TU (De Zwart and Posthuma 2005) curve reflects the seasonal variations of mixture effect, as well. Figure 3 shows that the TU and number of guest nights change together depending on time; their maximum values (TU, 22.75; and guest night, ~ 871,000 in August) are in high tourist seasons, while their minimum ones (TU, 0.01; and guest night, ~ 309,000 in November) are out of season. Although with only a difference of one order of magnitude, the fluctuation of mixture RQ shows similar seasonal changes in Xiangjiang River (Lin et al. 2018) like TU observing in our study area. Since the data used to calculate the mixture RQs are derived from RQs, they can be categorized as the same risk criteria. However, as already mentioned, TU has only one threshold. If its value greater than 1, it indicates a possible risk.

This is the first ERA based on changes in maxRQperiod values from a specific case study in Lake Balaton, which makes an effort to prove the harmful effect of summer tourist months on a freshwater lake.

## Summary

Season-dependent fluctuation of magnitude of risk is apparent (maxRQperiod, Fig. 2); therefore our hypothesis that the environmental risk increases during the holiday season in the study area, Lake Balaton, is proven. However, it must be noted that only 16 PhACs from the 42 present magnitude of the risk because they have available experimental ecotoxicological data (NOEC) applied to ERA. According to our results when considering all MAX RQs presented, the PhACs with at least medium risk level were caffeine, citalopram, diclofenac, E1, E2, E3, and EE2 in the study area during the period investigated. More attention should be paid to these 7 PhACs in the future in order to diagnose and predict their effects on aquatic ecosystems. The TU curve (Fig. 3) reflects the seasonal variations of mixture effect which correlate well with the change of maxRQperiods and the number of guest nights.

## Conclusions

The fluctuation of summed MEC, maxRQperiod, and TU suggested the possibility of harmful effects on aquatic ecosystems in the summer tourist season. Caffeine, citalopram, diclofenac, E1, E2, E3, and EE2 presented at least a medium risk at least once during the whole period of investigation in Lake Balaton, the largest shallow lake in Central Europe, based on MAX RQ results.

There is a real need for ongoing water quality monitoring and repeated toxicological testing for PhACs to ensure the real risk levels are understood. Besides, during our work, we found several discrepancies in raw ecotoxicological data; therefore, we propose to develop a unified PNEC database, including data regarding habitats, endpoints, and compounds, ensuring reliable and comparable results for ERA.

## Electronic supplementary material

ESM 1 (DOCX 738 kb)
