# Supplementary material for: Environmental risk assessment of pharmaceuticals at a seasonal holiday destination in the largest freshwater shallow lake in Central Europe
Source: Environ Sci Pollut Res Int. 2020 Jul 14;28(42):59233–43. doi: 10.1007/s11356-020-09747-4 (PMC8541981; doi:10.1007/s11356-020-09747-4)
Supplement: Supplementary file 1 — (DOCX 738 kb) [file 11356_2020_9747_MOESM1_ESM.docx]

**Supplamentary information**

**Environmental risk assessment of pharmaceuticals at a seasonal holiday destination in the largest freshwater shallow lake in Central Europe**

Eva Molnar, Gabor Maasz*, Zsolt Pirger

Adaptive Neuroethology Research Group, Department of Experimental Zoology, Balaton Limnological Institute, MTA Centre for Ecological Research, 8237 Tihany, Hungary

*Correspondence to Dr Gabor Maasz, Adaptive Neuroethology Research Group, Department of Experimental Zoology, Balaton Limnological Institute, MTA Centre for Ecological Research, 8237 Tihany, Hungary

E-mail: [maasz.gabor@okologia.mta.hu](mailto:maasz.gabor@okologia.mta.hu)

| PhACs' groups | **PhACs' name** | **LOQ** | June 2017 | | | | | | August 2017 | | | | | | November 2017 | | | | | | April 2018 | | | | | | June 2018 | | | | | | August 2018 | | | | | | October 2018 | | | | | | **MIN** | **MAX** | **FO** |
| --- | --- | --- | --- | --- | --- | --- | --- | --- | --- | --- | --- | --- | --- | --- | --- | --- | --- | --- | --- | --- | --- | --- | --- | --- | --- | --- | --- | --- | --- | --- | --- | --- | --- | --- | --- | --- | --- | --- | --- | --- | --- | --- | --- | --- | --- | --- | --- |
|  |  |  | Site 1 | Site 2 | Site 3 | Site 4 | Site 5 | Site 6 | Site 1 | Site 2 | Site 3 | Site 4 | Site 5 | Site 6 | Site 1 | Site 2 | Site 3 | Site 4 | Site 5 | Site 6 | Site 1 | Site 2 | Site 3 | Site 4 | Site 5 | Site 6 | Site 1 | Site 2 | Site 3 | Site 4 | Site 5 | Site 6 | Site 1 | Site 2 | Site 3 | Site 4 | Site 5 | Site 6 | Site 1 | Site 2 | Site 3 | Site 4 | Site 5 | Site 6 |  |  |  |
|  |  | **[ng/L]** | | | | | | | | | | | | | | | | | | | | | | | | | | | | | | | | | | | | | | | | | | | | | **[%]** |
| Antiepileptics | **carbamazepine** | **0,1** | 68,8 | 68,3 | 28,0 | 44,6 | 18,3 | 19,4 | 17,1 | 46,3 | 12,2 | 11,0 | 4,7 | 13,3 | 15,5 | 15,9 | 6,5 | 8,7 | 7,8 | 6,1 | 77,5 | 44,4 | 44,1 | 37,9 | 27,0 | 21,2 | - | 14,5 | - | 10,3 | 10,0 | 5,3 | 16,6 | 12,2 | 14,6 | 9,2 | 5,0 | 4,9 | 18,5 | 24,1 | 8,6 | 11,4 | 5,2 | 4,7 | **4,7** | **77,5** | **95,2** |
|  | **lamotrigine** | **5** | 7,1 | 5,7 | 7,0 | 8,6 | 6,0 | - | 28,3 | 162,2 | 29,9 | 15,5 | 17,6 | 17,0 | 22,1 | 22,0 | 17,1 | 18,6 | 22,0 | 19,7 | 18,6 | 16,4 | 23,5 | 19,3 | 33,4 | 16,6 | - | - | - | - | - | - | - | - | - | - | - | - | 55,4 | 54,9 | 30,0 | 34,7 | 23,3 | 21,0 | **5,7** | **162,2** | **69,0** |
| Antidepressants | **bupropion** | **0,5** | - | - | - | - | - | - | - | - | - | - | - | - | - | - | - | - | - | - | - | - | - | 6,6 | - | - | - | - | - | - | - | - | - | - | - | - | - | - | - | - | - | - | - | - | **6,6** | **6,6** | **2,4** |
|  | **citalopram** | **0,1** | - | - | - | - | 0,1 | - | - | 0,1 | - | - | - | 0,2 | - | - | - | - | - | - | - | - | - | 2,4 | - | - | - | - | - | - | - | - | - | - | - | - | - | - | - | - | - | - | - | - | **0,1** | **2,4** | **9,5** |
|  | **clozapine** | **0,1** | - | 0,4 | - | 0,2 | - | 0,5 | - | 0,3 | - | 0,6 | 0,2 | - | - | - | - | - | - | - | - | - | - | - | - | - | - | - | - | - | - | - | - | - | - | - | - | - | - | - | - | 0,6 | - | - | **0,2** | **0,6** | **16,7** |
|  | **fluoxetine** | **0,5** | 0,5 | - | 1,7 | - | 1,4 | - | - | - | - | - | - | - | - | - | - | - | - | - | - | - | - | - | - | - | - | - | - | - | - | - | - | - | - | - | - | - | - | - | - | - | - | - | **0,5** | **1,7** | **7,1** |
|  | **mirtazapine** | **0,1** | 0,4 | - | - | 0,2 | - | 0,5 | - | 0,2 | - | 0,5 | - | - | - | - | - | - | 0,2 | - | - | - | - | - | - | - | - | - | - | - | - | - | 0,1 | - | - | - | - | 0,2 | - | 0,1 | - | 0,8 | - | - | **0,1** | **0,8** | **23,8** |
|  | **olanzapine** | **5** | - | - | - | - | - | - | - | - | - | - | - | - | - | 11,8 | - | - | - | - | - | - | - | - | - | - | - | - | - | - | - | - | - | - | - | - | - | - | - | - | - | - | - | - | **11,8** | **11,8** | **2,4** |
|  | **quetiapine** | **0,1** | - | - | - | - | - | 0,1 | - | - | 0,1 | - | - | - | - | - | - | - | - | - | - | - | - | - | - | - | - | - | - | - | - | - | - | - | - | - | - | - | - | - | - | - | - | - | **0,1** | **0,1** | **4,8** |
|  | **tiapride** | **0,1** | 2,5 | 0,5 | 0,7 | 0,8 | 0,6 | 0,2 | 6,7 | 14,4 | 2,3 | 6,2 | 3,0 | 1,1 | 11,8 | 4,6 | 1,4 | 2,4 | 1,5 | 0,6 | 12,0 | 0,4 | 1,3 | 0,1 | - | - | 0,6 | - | - | - | - | - | 0,2 | - | 1,3 | - | - | 1,1 | 1,3 | 0,6 | - | 0,9 | - | - | **0,1** | **14,4** | **69,0** |
| Anxiolytics | **alprazolam** | **0,1** | 0,1 | 0,1 | - | - | 0,1 | - | 0,2 | 0,9 | 0,2 | 0,2 | 0,2 | 0,2 | 0,2 | 0,3 | 0,3 | 0,2 | 0,2 | 0,2 | 0,7 | - | - | 0,2 | - | 0,1 | - | - | - | - | - | - | - | - | - | - | - | - | - | 0,5 | - | 0,3 | - | - | **0,1** | **0,9** | **47,6** |
|  | **buspirone** | **0,1** | - | - | - | - | - | - | - | 0,1 | - | - | - | 0,1 | - | - | - | - | - | - | 5,9 | - | - | - | - | - | - | - | - | - | - | - | - | - | - | - | - | - | - | - | - | - | - | - | **0,1** | **5,9** | **7,1** |
|  | **diazepam** | **0,1** | - | - | - | - | - | - | - | - | - | - | - | - | - | - | - | - | - | - | 0,3 | - | - | - | - | - | - | - | - | - | - | - | - | - | - | - | - | - | - | - | - | - | - | - | **0,3** | **0,3** | **2,4** |
|  | **midazolam** | **0,1** | - | - | - | - | - | - | - | - | - | - | - | - | - | - | - | - | - | - | 1,7 | - | - | - | - | - | - | - | - | - | - | - | - | - | - | - | - | - | - | - | - | - | - | - | **1,7** | **1,7** | **2,4** |
|  | **nordiazepam** | **0,1** | - | 1,4 | - | - | - | - | - | - | - | - | - | - | - | - | - | - | 0,4 | - | - | - | - | - | - | - | - | - | - | - | - | - | - | - | - | - | - | - | - | - | - | - | - | - | **0,4** | **1,4** | **4,8** |
|  | **zolpidem** | **0,01** | - | - | - | - | - | - | - | - | - | - | - | - | - | - | - | - | - | - | 0,2 | - | - | - | - | - | - | - | - | - | - | - | - | - | - | - | - | - | - | - | - | - | - | - | **0,2** | **0,2** | **2,4** |
|  | **bisoprolol** | **0,5** | - | - | 6,3 | 0,7 | 2,1 | - | 0,9 | 2,9 | 0,5 | 2,9 | 16,7 | 14,6 | 3,1 | 1,5 | 2,8 | 1,5 | 1,4 | 3,4 | - | 0,5 | - | - | - | - | - | - | - | - | 1,9 | 2,0 | - | - | 1,9 | - | 1,5 | 2,5 | - | - | 1,5 | - | 1,2 | 2,7 | **0,5** | **16,7** | **57,1** |
|  | **losartan** | **0,1** | - | - | - | - | - | - | - | - | - | - | - | - | - | - | - | - | - | - | 0,8 | 0,1 | - | - | - | - | - | - | - | - | - | - | - | - | - | - | - | - | 0,2 | - | - | 0,2 | - | - | **0,1** | **0,8** | **9,5** |
|  | **metoprolol** | **0,1** | - | - | - | - | - | - | - | 5,1 | - | - | - | - | - | - | - | - | - | - | - | 1,2 | - | - | - | - | - | - | - | - | 0,3 | - | - | - | - | - | - | - | - | 1,3 | - | - | - | - | **0,3** | **5,1** | **9,5** |
|  | **perindopril** | **0,1** | 0,9 | 1,2 | 0,3 | 0,5 | 0,4 | 0,1 | 1,9 | 17,7 | 0,8 | 1,2 | 2,2 | 0,9 | 1,6 | 3,8 | 0,6 | 0,5 | 1,0 | 0,5 | 2,1 | 1,9 | 0,8 | 0,8 | - | - | - | - | - | - | - | - | - | - | - | - | - | - | 0,9 | 6,1 | 0,4 | 0,7 | 0,1 | - | **0,1** | **17,7** | **64,3** |
|  | **verapamil** | **0,05** | - | - | 0,5 | 0,1 | 0,1 | - | 1,3 | 0,4 | 0,1 | 2,0 | 27,1 | 6,7 | - | - | - | - | - | - | 1,4 | - | - | - | - | - | - | - | - | - | - | - | - | - | - | - | - | - | - | - | - | - | - | - | **0,1** | **27,1** | **23,8** |
| Hormones | **bE2** | **0,05** | 4,0 | 2,9 | 19,6 | 3,4 | 17,0 | 3,7 | 0,2 | - | - | - | - | 0,1 | - | - | - | - | - | - | - | - | 0,2 | - | - | - | - | - | - | 0,8 | - | 3,0 | - | - | - | - | - | - | - | - | 0,1 | - | - | - | **0,1** | **19,6** | **28,6** |
|  | **E1** | **0,05** | 5,5 | 3,6 | 1,0 | 1,0 | 0,4 | - | 0,9 | 0,9 | 1,2 | 0,5 | 0,4 | 0,5 | 0,4 | 0,3 | 0,2 | 0,3 | 0,2 | 0,2 | 0,5 | 0,3 | 0,5 | 0,2 | 0,2 | - | - | - | - | - | - | - | 1,1 | 0,6 | 1,1 | 1,8 | 0,3 | 0,5 | - | - | 0,4 | - | 0,1 | - | **0,1** | **5,5** | **71,4** |
|  | **E3** | **0,05** | - | - | - | - | 0,1 | - | - | - | - | - | - | 0,1 | - | - | - | - | - | - | - | - | - | - | - | - | - | - | - | - | - | - | - | - | - | - | - | - | - | - | - | - | - | - | **0,1** | **0,1** | **4,8** |
|  | **EE2** | **0,05** | - | - | - | - | - | - | - | - | - | - | - | - | 0,2 | - | - | - | - | - | - | - | - | - | - | - | - | - | - | - | - | - | - | - | - | - | - | - | - | - | - | - | - | - | **0,2** | **0,2** | **2,4** |
|  | **levonorgestrel** | **1** | - | - | - | - | - | - | - | - | - | - | - | - | - | - | - | - | - | - | - | - | - | - | 1,8 | - | - | - | - | - | - | - | - | - | - | - | - | - | - | - | - | - | 2,3 | - | **1,8** | **2,3** | **4,8** |
|  | **progesterone** | **0,5** | - | 0,9 | 0,7 | 1,0 | - | - | - | 1,0 | - | 0,6 | 1,1 | 1,3 | - | - | - | - | - | - | - | - | - | 1,1 | - | - | - | - | - | - | - | - | - | - | - | - | - | - | - | - | - | - | - | - | **0,6** | **1,3** | **19,0** |
|  | **testosterone** | **0,5** | - | - | - | - | - | - | - | - | - | - | - | - | - | - | - | - | - | - | - | - | 1,1 | - | - | - | - | - | - | - | - | - | - | - | - | - | - | - | - | - | - | - | - | - | **1,1** | **1,1** | **2,4** |
| Local anesthetics | **lidocaine** | **0,1** | 0,4 | 0,5 | 42,2 | 0,3 | 27,8 | - | 1,1 | 3,6 | 1,0 | 0,5 | 1,4 | 1,5 | 1,8 | 0,5 | 0,1 | 0,3 | 0,2 | 0,4 | 5,8 | - | - | - | 0,3 | - | 2,4 | 10,9 | 3,7 | 4,5 | 6,2 | 5,2 | 1,3 | - | 2,1 | - | 2,0 | 2,4 | 1,1 | 0,7 | - | 1,5 | - | 0,3 | **0,1** | **42,2** | **78,6** |
|  | **tetracaine** | **0,1** | - | - | 1,2 | - | 0,2 | - | - | - | - | - | - | - | - | - | - | - | - | - | - | - | - | - | - | - | - | - | - | - | - | - | - | - | - | - | - | - | - | - | - | - | - | - | **0,2** | **1,2** | **4,8** |
| NSAIDs | **diclofenac** | **0,5** | - | - | - | - | - | - | - | - | - | - | - | - | - | - | - | - | - | - | - | - | - | - | - | - | 12,9 | 53,9 | - | 59,1 | 8,7 | 5,3 | 419,4 | 25,3 | 6,7 | 5,6 | 13,2 | - | 196,7 | 163,9 | 13,0 | 324,1 | 32,4 | 101,5 | **5,3** | **419,4** | **38,1** |
|  | **naproxen** | **0,1** | - | - | - | - | - | - | - | - | - | - | 2,2 | - | - | - | - | - | - | - | - | - | - | - | - | - | - | - | - | - | - | - | - | - | - | - | - | - | 15,5 | - | 27,7 | - | - | 10,6 | **2,2** | **27,7** | **9,5** |
| Opioids | **methadone** | **0,02** | - | - | - | - | - | - | - | - | - | - | - | - | - | - | - | - | - | - | 0,6 | - | - | - | - | - | - | - | - | - | - | - | - | - | - | - | - | - | - | - | - | - | - | - | **0,6** | **0,6** | **2,4** |
|  | **tramadol** | **0,1** | 0,3 | 0,5 | 0,3 | 0,3 | 0,2 | - | 1,5 | 6,1 | 0,7 | 0,7 | 0,8 | 1,0 | 1,5 | 0,8 | 0,4 | 0,4 | 0,6 | 0,3 | 3,0 | 0,9 | 1,0 | 0,8 | 0,4 | 0,2 | 0,9 | 0,7 | 0,6 | 0,4 | 0,4 | 0,2 | 0,8 | 0,6 | - | 0,8 | 0,5 | - | 0,8 | 0,6 | 0,3 | 0,6 | 0,2 | 0,2 | **0,2** | **6,1** | **92,9** |
| Others | **ketamin** | **0,5** | - | - | - | - | - | - | - | - | - | - | 2,5 | 8,8 | - | - | - | - | - | - | - | - | - | - | - | - | - | - | - | - | - | - | - | - | - | - | - | - | - | - | - | - | - | - | **2,5** | **8,8** | **4,8** |
|  | **atropine** | **0,05** | - | - | - | - | - | - | - | - | 0,4 | - | - | - | - | - | - | - | - | - | - | 2,2 | - | - | - | - | - | - | - | - | - | - | - | - | - | - | - | - | - | - | - | - | - | - | **0,4** | **2,2** | **4,8** |
|  | **caffeine** | **10** | - | - | - | - | - | - | - | 15,6 | - | - | 79,8 | 89,9 | - | - | - | - | - | - | - | - | - | - | - | - | 545,8 | 939,4 | 398,4 | 1179,6 | 1192,4 | 1388,0 | 2675,1 | 160,4 | 274,5 | 166,5 | 426,2 | 656,0 | 1695,6 | 1726,7 | 605,9 | 2415,9 | 454,6 | - | **15,6** | **2675,1** | **47,6** |
|  | **theophyllin** | **10** | - | - | - | - | - | - | - | - | - | - | - | - | - | - | - | - | - | - | - | - | - | - | - | - | - | - | - | - | - | - | - | - | - | - | - | - | - | 29,3 | - | 51,7 | 28,9 | 59,6 | **28,9** | **59,6** | **9,5** |
| Hallucinogens | **benzoyl-ecgonine** | **0,1** | - | - | - | - | - | - | - | 0,2 | - | - | 0,8 | 2,3 | - | - | - | - | - | - | - | - | - | - | - | - | - | - | - | - | - | - | - | - | - | - | - | - | - | 0,1 | - | - | - | - | **0,1** | **2,3** | **9,5** |
|  | **cocaine** | **0,05** | - | - | - | - | - | - | - | - | - | - | 0,2 | 0,1 | - | - | - | - | - | - | - | - | - | - | - | - | - | - | - | - | - | - | - | - | - | - | - | - | - | - | - | - | - | - | **0,1** | **0,2** | **4,8** |
|  | **MDMA (Ecstasy)** | **2** | - | - | - | - | - | - | - | - | - | - | - | 9,2 | - | - | - | - | - | - | - | - | - | - | - | - | - | - | - | - | - | - | - | - | - | - | - | - | - | - | - | - | - | - | **9,2** | **9,2** | **2,4** |
| Sedatives | **barbital** | **10** | - | - | - | - | - | - | - | - | - | - | - | - | - | - | - | - | - | - | - | - | - | - | - | - | - | 94,8 | - | - | - | - | - | - | - | - | - | - | - | - | - | - | - | - | **94,8** | **94,8** | **2,4** |

**Supplementary Table 1** - Concentration levels of 42 detected PhCAs in 6 sites of Lake Balaton in 7 investigated periods with their limit of quantification (LOQ), minimum (MIN), maximum (MAX) and frequency of occurrence (FO) data, - represents <LOQ

**Supplementary Fig. 1** - Seasonal fluctuation of summed MEC (sumMEC) and number of guest nights in Lake Balaton in the investigated months

| **PhACs** | **Lake Balaton (1-6)** | | | | | | | | | | | | | | | |
| --- | --- | --- | --- | --- | --- | --- | --- | --- | --- | --- | --- | --- | --- | --- | --- | --- |
|  | **June 2017** | | **August 2017** | | **November 2017** | | **April 2018** | | **June 2018** | | **August 2018** | | **October 2018** | | **June 2017 - October 2018** | |
|  | **MEC** | **maxRQ** | **MEC** | **maxRQ** | **MEC** | **maxRQ** | **MEC]** | **maxRQ** | **MEC** | **maxRQ** | **MEC** | **maxRQ** | **MEC** | **maxRQ** | **MAX RQ** | **Level of risk** |
|  | **[ng/L]** |  | **[ng/L]** |  | **[ng/L]** |  | **[ng/L]** |  | **[ng/L]** |  | **[ng/L]** |  | **[ng/L]** |  |  |  |
| **E2** | 1.96E+01 | 4.45E-01 | 2.00E-01 | 4.55E-03 | <LOQ | - | 1.95E-01 | 4.43E-03 | 3.00E+00 | 6.81E-02 | <LOQ | - | 6.50E-02 | 1.48E-03 | 4.45E-01 | medium |
|  |  | 4.90E+01 |  | 5.00E-01 |  |  |  | 4.88E-01 |  | 7.50E+00 |  |  |  | 1.63E-01 | 4.90E+01 | high |
|  |  | 1.96E+02 |  | 2.00E+00 |  |  |  | 1.95E+00 |  | 3.00E+01 |  |  |  | 6.50E-01 | 1.96E+02 | high |
|  |  | 2.68E+01 |  | 2.74E-01 |  |  |  | 2.67E-01 |  | 4.11E+00 |  |  |  | 8.90E-02 | 2.68E+01 | high |
|  |  | 1.96E+01 |  | 2.00E-01 |  |  |  | 1.95E-01 |  | 3.00E+00 |  |  |  | 6.50E-02 | 1.96E+01 | high |
|  |  | 9.80E+00 |  | 1.00E-01 |  |  |  | 9.75E-02 |  | 1.50E+00 |  |  |  | 3.25E-02 | 9.80E+00 | high |
| **caffeine** | <LOQ | - | 8.99E+01 | 1.31E-02 | <LOQ | - | <LOQ | - | 1.39E+03 | 2.03E-01 | 2.68E+03 | 3.90E-01 | 2.42E+03 | 3.53E-01 | 3.90E-01 | medium |
|  |  |  |  | 3.88E-02 |  |  |  |  |  | 6.00E-01 |  | 1.16E+00 |  | 1.04E+00 | 1.16E+00 | high |
| tramadol | 4.90E-01 | 1.53E-02 | 6.10E+00 | 1.91E-01 | 1.54E+00 | 4.81E-02 | 3.02E+00 | 9.44E-02 | 9.02E-01 | 2.82E-02 | 8.34E-01 | 2.60E-02 | 7.94E-01 | 2.48E-02 | 1.91E-01 | medium |
| **diclofenac** | <LOQ | - | <LOQ | - | <LOQ | - | <LOQ | - | 5.91E+01 | 1.40E-02 | 4.19E+02 | 9.89E-02 | 3.24E+02 | 7.65E-02 | 9.89E-02 | low |
|  |  |  |  |  |  |  |  |  |  | 1.18E+00 |  | 8.38E+00 |  | 6.48E+00 | 8.38E+00 | high |
|  |  |  |  |  |  |  |  |  |  | 5.58E+00 |  | 3.95E+01 |  | 3.06E+01 | 3.95E+01 | high |
| **E1** | 5.52E+00 | 7.46E-02 | 1.23E+00 | 1.66E-02 | 4.30E-01 | 5.81E-03 | 5.10E-01 | 6.89E-03 | <LOQ | - | 1.81E+00 | 2.44E-02 | 4.25E-01 | 5.74E-03 | 7.46E-02 | low |
|  |  | 1.53E+00 |  | 3.42E-01 |  | 1.19E-01 |  | 1.42E-01 |  |  |  | 5.03E-01 |  | 1.18E-01 | 1.53E+00 | high |
|  |  | 5.52E+00 |  | 1.23E+00 |  | 4.30E-01 |  | 5.10E-01 |  |  |  | 1.81E+00 |  | 4.25E-01 | 5.52E+00 | high |
| theophylline | <LOQ | - | <LOQ | - | <LOQ | - | <LOQ | - | <LOQ | - | <LOQ | - | 5.96E+01 | 5.96E-02 | 5.96E-02 | low |
| MDMA | <LOQ | - | 9.15E+00 | 4.24E-02 | <LOQ | - | <LOQ | - | <LOQ | - | <LOQ | - | <LOQ | - | 4.24E-02 | low |
| lidocaine | 4.22E+01 | 1.62E-02 | 3.55E+00 | 1.36E-03 | 1.82E+00 | 6.98E-04 | 5.81E+00 | 2.22E-03 | 1.09E+01 | 4.16E-03 | 2.42E+00 | 9.27E-04 | 1.50E+00 | 5.75E-04 | 1.62E-02 | low |
| **carbamazepine** | 6.88E+01 | 1.08E-02 | 4.63E+01 | 7.28E-03 | 1.59E+01 | 2.50E-03 | 7.75E+01 | 1.22E-02 | 1.45E+01 | 2.28E-03 | 1.66E+01 | 2.61E-03 | 2.41E+01 | 3.79E-03 | 1.22E-02 | low |
|  |  | 6.88E-03 |  | 4.63E-03 |  | 1.59E-03 |  | 7.75E-03 |  | 1.45E-03 |  | 1.66E-03 |  | 2.41E-03 | 7.75E-03 | negligible |
| ketamin | <LOQ | - | 8.79E+00 | 1.02E-02 | <LOQ | - | <LOQ | - | <LOQ | - | <LOQ | - | <LOQ | - | 1.02E-02 | low |
| **fluoxetine** | 1.68E+00 | 9.44E-03 | <LOQ | - | <LOQ | - | <LOQ | - | <LOQ | - | <LOQ | - | <LOQ | - | 9.44E-03 | negligible |
|  |  | 1.56E-03 |  |  |  |  |  |  |  |  |  |  |  |  | 1.56E-03 | negligible |
| **E3** | 1.00E-01 | 6.67E-03 | 1.30E-01 | 8.67E-03 | <LOQ | - | <LOQ | - | <LOQ | - | <LOQ | - | <LOQ | - | 8.67E-03 | negligible |
|  |  | 2.15E-01 |  | 2.80E-01 |  |  |  |  |  |  |  |  |  |  | 2.80E-01 | medium |
| bupropion | <LOQ | - | <LOQ | - | <LOQ | - | 6.59E+00 | 6.94E-03 | <LOQ | - | <LOQ | - | <LOQ | - | 6.94E-03 | negligible |
| midazolam | <LOQ | - | <LOQ | - | <LOQ | - | 1.74E+00 | 6.00E-03 | <LOQ | - | <LOQ | - | <LOQ | - | 6.00E-03 | negligible |
| bisoprolol | 6.28E+00 | 1.99E-03 | 1.67E+01 | 5.29E-03 | 3.35E+00 | 1.06E-03 | 5.35E-01 | 1.70E-04 | 1.98E+00 | 6.26E-04 | 2.50E+00 | 7.92E-04 | 2.70E+00 | 8.56E-04 | 5.29E-03 | negligible |
| **EE2** | <LOQ | - | <LOQ | - | 1.80E-01 | 4.50E-03 | <LOQ | - | <LOQ | - | <LOQ | - | <LOQ | - | 4.50E-03 | negligible |
|  |  |  |  |  |  | 4.09E-01 |  |  |  |  |  |  |  |  | 4.09E-01 | medium |
| levonorgestrel | <LOQ | - | <LOQ | - | <LOQ | - | 1.84E+00 | 3.31E-03 | <LOQ | - | <LOQ | - | 2.31E+00 | 4.16E-03 | 4.16E-03 | negligible |
| **citalopram** | 1.30E-01 | 2.05E-04 | 2.00E-01 | 3.15E-04 | <LOQ | - | 2.44E+00 | 3.83E-03 | <LOQ | - | <LOQ | - | <LOQ | - | 3.83E-03 | negligible |
|  |  | 1.30E-02 |  | 2.00E-02 |  |  |  | 2.44E-01 |  |  |  |  |  |  | 2.44E-01 | medium |
| buspirone | <LOQ | - | 1.20E-01 | 4.61E-05 | <LOQ | - | 5.94E+00 | 2.28E-03 | <LOQ | - | <LOQ | - | <LOQ | - | 2.28E-03 | negligible |
| naproxen | <LOQ | - | 2.19E+00 | 1.45E-04 | <LOQ | - | <LOQ | - | <LOQ | - | <LOQ | - | 2.77E+01 | 1.83E-03 | 1.83E-03 | negligible |
| **progesterone** | 9.60E-01 | 1.31E-03 | 1.31E+00 | 1.79E-03 | <LOQ | - | 1.13E+00 | 1.54E-03 | <LOQ | - | <LOQ | - | <LOQ | - | 1.79E-03 | negligible |
|  |  | 9.60E-04 |  | 1.31E-03 |  |  |  | 1.13E-03 |  |  |  |  |  |  | 1.31E-03 | negligible |
| alprazolam | 1.40E-01 | 2.76E-04 | 8.80E-01 | 1.73E-03 | 3.30E-01 | 6.50E-04 | 7.05E-01 | 1.39E-03 | <LOQ | - | <LOQ | - | 4.52E-01 | 8.89E-04 | 1.73E-03 | negligible |
| tiapride | 2.53E+00 | 2.90E-04 | 1.44E+01 | 1.66E-03 | 1.18E+01 | 1.36E-03 | 1.20E+01 | 1.38E-03 | 5.94E-01 | 6.81E-05 | 1.25E+00 | 1.44E-04 | 1.33E+00 | 1.53E-04 | 1.66E-03 | negligible |
| tetracaine | 1.18E+00 | 1.58E-03 | <LOQ | - | <LOQ | - | <LOQ | - | <LOQ | - | <LOQ | - | <LOQ | - | 1.58E-03 | negligible |
| nordiazepam | 1.39E+00 | 1.17E-03 | <LOQ | - | 3.80E-01 | 3.20E-04 | <LOQ | - | <LOQ | - | <LOQ | - | <LOQ | - | 1.17E-03 | negligible |
| atropine | <LOQ | - | 4.10E-01 | 1.54E-04 | <LOQ | - | 2.20E+00 | 8.28E-04 | <LOQ | - | <LOQ | - | <LOQ | - | 8.28E-04 | negligible |
| **testosterone** | <LOQ | - | <LOQ | - | <LOQ | - | 1.09E+00 | 7.62E-04 | <LOQ | - | <LOQ | - | <LOQ | - | 7.62E-04 | negligible |
|  |  |  |  |  |  |  |  | 1.09E-03 |  |  |  |  |  |  | 1.09E-03 | negligible |
| verapamil | 5.30E-01 | 1.47E-05 | 2.71E+01 | 7.54E-04 | <LOQ | - | 1.43E+00 | 3.96E-05 | <LOQ | - | <LOQ | - | <LOQ | - | 7.54E-04 | negligible |
| losartan | <LOQ | - | <LOQ | - | <LOQ | - | 8.45E-01 | 4.45E-04 | <LOQ | - | <LOQ | - | 2.18E-01 | 1.14E-04 | 4.45E-04 | negligible |
| zolpidem | <LOQ | - | <LOQ | - | <LOQ | - | 2.20E-01 | 4.24E-04 | <LOQ | - | <LOQ | - | <LOQ | - | 4.24E-04 | negligible |
| **clozapine** | 5.40E-01 | 3.68E-04 | 5.50E-01 | 3.75E-04 | <LOQ | - | <LOQ | - | <LOQ | - | <LOQ | - | 5.54E-01 | 3.77E-04 | 3.77E-04 | negligible |
|  |  | 1.89E-03 |  | 1.93E-03 |  |  |  |  |  |  |  |  |  | 1.94E-03 | 1.94E-03 | negligible |
| **diazepam** | <LOQ | - | <LOQ | - | <LOQ | - | 2.50E-01 | 1.76E-04 | <LOQ | - | <LOQ | - | <LOQ | - | 1.76E-04 | negligible |
|  |  |  |  |  |  |  |  | 9.62E-05 |  |  |  |  |  |  | 9.62E-05 | negligible |
| olanzapine | <LOQ | - | <LOQ | - | 1.18E+01 | 8.36E-05 | <LOQ | - | <LOQ | - | <LOQ | - | <LOQ | - | 8.36E-05 | negligible |
| **metoprolol** | <LOQ | - | 5.08E+00 | 8.26E-05 | <LOQ | - | 1.17E+00 | 1.90E-05 | 2.64E-01 | 4.28E-06 | <LOQ | - | 1.25E+00 | 2.04E-05 | 8.26E-05 | negligible |
| barbital | <LOQ | - | <LOQ | - | <LOQ | - | <LOQ | - | 9.48E+01 | 8.16E-05 | <LOQ | - | <LOQ | - | 8.16E-05 | negligible |
| cocaine | <LOQ | - | 1.60E-01 | 7.01E-05 | <LOQ | - | <LOQ | - | <LOQ | - | <LOQ | - | <LOQ | - | 7.01E-05 | negligible |
| mirtazapine | 5.10E-01 | 1.59E-05 | 5.30E-01 | 1.66E-05 | 1.90E-01 | 5.94E-06 | <LOQ | - | <LOQ | - | 2.34E-01 | 7.31E-06 | 7.65E-01 | 2.39E-05 | 2.39E-05 | negligible |
| perindopril | 1.24E+00 | 1.25E-06 | 1.77E+01 | 1.79E-05 | 3.79E+00 | 3.83E-06 | 2.11E+00 | 2.13E-06 | <LOQ | - | <LOQ | - | 6.15E+00 | 6.21E-06 | 1.79E-05 | negligible |
| methadone | <LOQ | - | <LOQ | - | <LOQ | - | 6.40E-01 | 1.68E-05 | <LOQ | - | <LOQ | - | <LOQ | - | 1.68E-05 | negligible |
| **quetiapine** | 1.20E-01 | 1.20E-05 | 1.10E-01 | 1.10E-05 | <LOQ | - | <LOQ | - | <LOQ | - | <LOQ | - | <LOQ | - | 1.20E-05 | negligible |
| **lamotrigine** | 8.57E+00 | 5.71E-08 | 1.62E+02 | 1.08E-06 | 2.21E+01 | 1.47E-07 | 3.34E+01 | 2.23E-07 | <LOQ | - | <LOQ | - | 5.54E+01 | 3.69E-07 | 1.08E-06 | negligible |
| benzoylecgonine | <LOQ | - | 2.33E+00 | 3.42E-07 | <LOQ | - | <LOQ | - | <LOQ | - | <LOQ | - | <LOQ | - | 3.42E-07 | negligible |

**Supplementary Table 2 -** MEC data (in ng/L), calculated maxRQ, and MAX RQ values of 42 PhACs, as well as risk levels of study area, in the periods investigated (LOQ = limit of quantitation)

**
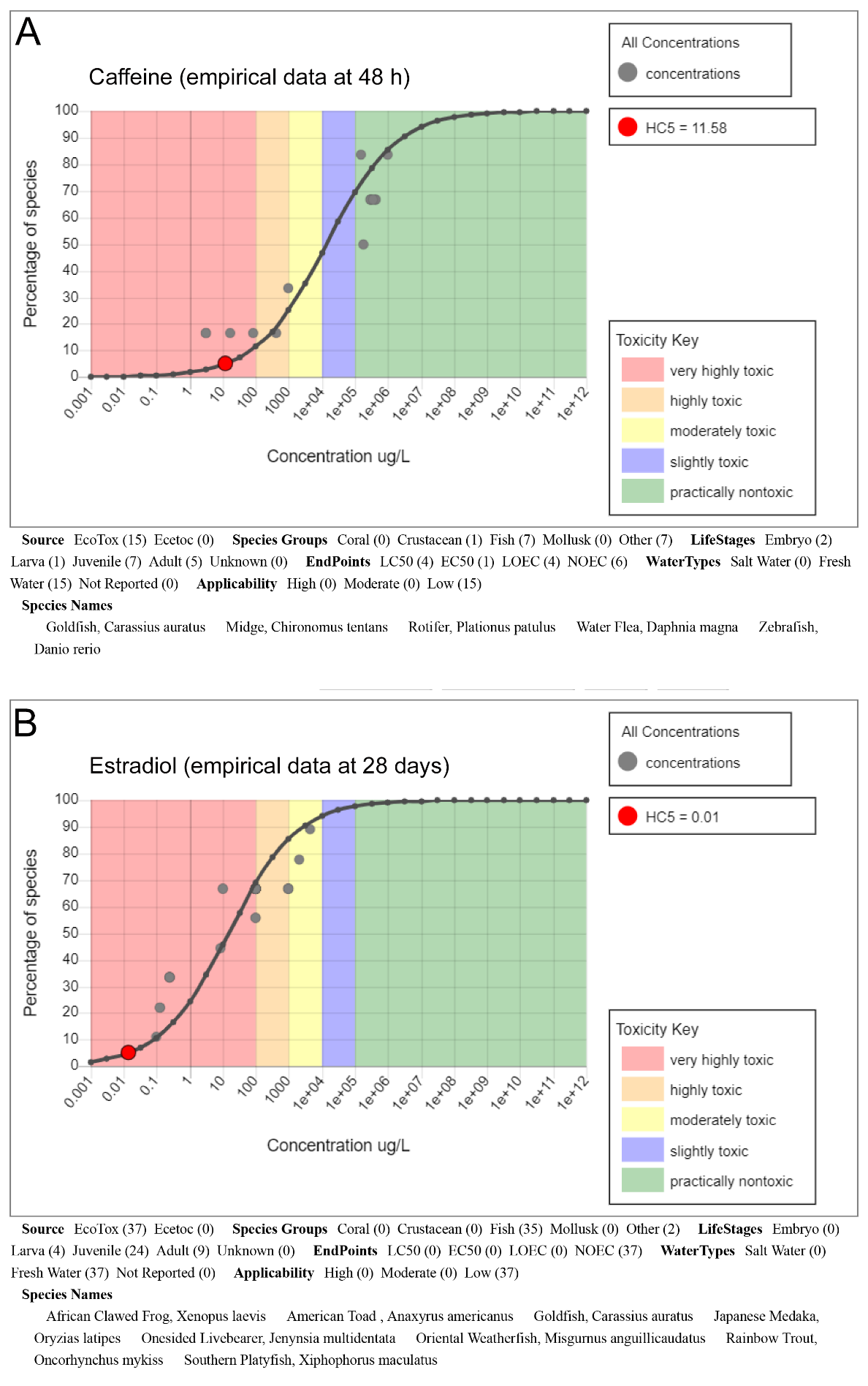
**

**Supplementary Fig. 2** - SSD curves of caffeine (A) and estradiol (B) derived from CAFE database with search conditions. HC5 – represents hazard concentration in case of 5% of the species in the SSD exhibit an effect
